# Surface Enhanced Nonlinear Raman Processes for Advanced Vibrational Probing

**DOI:** 10.1021/acsnano.4c07508

**Published:** 2024-08-01

**Authors:** Janina Kneipp, Katrin Kneipp

**Affiliations:** Humboldt-Universität zu Berlin, Department of Chemistry, Brook-Taylor-Str. 2, 12489 Berlin, Germany

**Keywords:** surface enhanced Raman scattering (SERS), plasmon, surface enhanced hyper Raman scattering (SEHRS), surface enhanced coherent anti-Stokes Raman scattering (SECARS), surface enhanced stimulated Raman scattering (SESRS), surface enhanced pumped anti-Stokes Raman scattering (SEPARS), plasmon-molecule interaction, composite nanomaterials

## Abstract

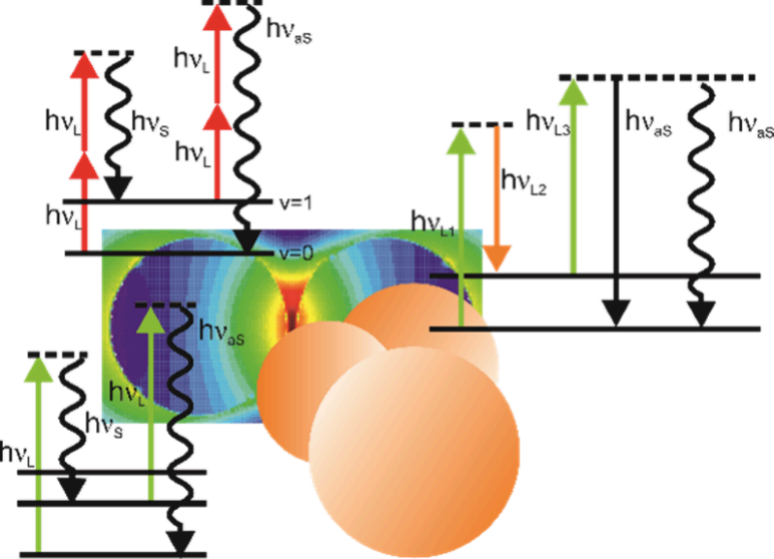

Surface enhanced Raman scattering (SERS) is not restricted to the well-known one-photon excited spontaneous Raman process that gives information on molecular composition, structure, and interaction through vibrational probing with high sensitivity. The enhancement mainly originates in high local fields, specifically those provided by localized surface plasmon resonances of metal nanostructures. High local fields can particularly support nonlinear Raman scattering, as it depends on the fields to higher powers. By revealing plasmon-molecule interactions, nonlinear Raman processes provide a very sensitive access to the properties of metal nanomaterials and their interfaces with molecules and other materials. This Perspective discusses plasmon-enhanced spontaneous and coherent nonlinear Raman scattering with the aim of identifying advantages that lead to an advanced vibrational characterization of such systems. The discussion will highlight the aspects of vibrational information that can be gained based on specific advantages of different incoherent and coherent Raman scattering and their surface enhancement. While the incoherent process of surface enhanced hyper Raman scattering (SEHRS) gives highly selective and spectral information complementary to SERS, the incoherent process of surface enhanced pumped anti-Stokes Raman scattering (SEPARS) can help to infer effective nonresonant SERS cross sections and allows to see “hot” vibrational transitions. Surface enhanced coherent anti-Stokes Raman scattering (SECARS) and surface enhanced stimulated Raman scattering (SESRS) combine the advantages of high local fields and coherence, which gives rise to high detection sensitivity and offers possibilities to explore molecule-plasmon interactions for a comprehensive characterization of composite and hybrid structures in materials research, catalysis, and nanobiophotonics.

## Introduction

Vibrational spectra, obtained by infrared absorption or Raman scattering, provide direct access to chemical and physical properties of molecules. During Raman scattering, light is inelastically scattered on the vibrational quantum states. In this process, photons may lose energy to, or gain it from, vibrational excitations. A change Δ*E* in the vibrational energy of the molecule must produce a change in the frequency of the scattered light, visible as a frequency shift relative to the excitation. By this Raman shift, vibrational information, which occurs at energies in the infrared and terahertz range, can be obtained in the visible or near-infrared (NIR) range. Raman scattering is a very weak effect, with typical Raman cross sections between 10^–30^ to 10^–25^ cm^2^ per molecule, with the larger values occurring only under favorable resonance Raman conditions when the excitation energy matches that of an electronic transition in the molecule. The small Raman scattering signals are a particular drawback when the number of molecules is small. However, by surface enhanced Raman scattering (SERS),^1^ Raman signals can be dramatically boosted and can achieve single molecule sensitivity.^2,3^

The increase in sensitivity in vibrational probing and the selectivity of a SERS experiment have led to the wide application of the effect, and have improved our understanding of molecule-(metal)surface interactions in many fields of chemistry and nanoscience, including catalysis, materials research, and nanobiophotonics.^4^ For several decades, strong evidence has been provided that enhanced local fields in the close vicinity of silver and gold nanostructures due to resonances with plasmonic excitations must play an important role in SERS.^5^ On the other hand, some SERS experiments indicate an additional enhancement up to 3 orders of magnitude when molecules directly interact with the surface of a nanostructure. Charge transfer between molecule and metal is considered as most likely basic process for these “chemical” contributions to SERS.^6^ The chemical enhancement is highly molecule specific and refers to the properties of the metal-molecule system, which can be different from those of the free molecule.

Particularly the observation of strong nonlinear SERS effects provides evidence that enhanced local fields are the key effect in SERS. Theory has shown and discussed high local fields for metal nanostructures, such as aggregates of nanoparticles, fractal structures, semicontinuous random metal films, and tailored nanostructures, where bright and also dark modes of localized surface plasmon resonances play a role.^7^ High local optical fields exist in small gaps in metal nanostructures and are restricted to extremely small volumes in nanometer dimensions of so-called hot spots. SERS studies on dimers of plasmonic nanoparticles have shown an increase of SERS enhancement with decreasing gap widths. A decrease of the enhancement sets on for dimers with gaps in the subnanometer range, in contrast to the behavior of “classical” plasmonic dimers, which might indicate the onset of quantum effects due to such extremely narrow gaps in plasmonic structures.^8^ In a more recent optomechanics approach, SERS is considered in a quantum description of a molecule in a cavity.^9^ In a combined system of metal nanoparticles and molecules or another material, vibrational probing that relies on localized surface plasmon resonances such as SERS can provide a direct access to both, the structure and interaction of the molecule, as well as the plasmonic properties of the metal structure. As we will discuss, nonlinear Raman probing can become particularly sensitive to such properties.

The electromagnetic enhancement of the excitation field and the scattering field in a Raman process can be described by a frequency-dependent field enhancement factor *A*(ν) for each field. Together with the chemical enhancement, represented by the increased Raman cross section σ_*ads*_^*RS*^ of the molecule adsorbed to the surface of the nanostructure, the number of SERS photons in a spontaneous Raman process *n*_*SERS*_ is
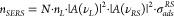
1with *ν*_*L*_ and *ν*_*RS*_ being the excitation laser and Raman Stokes (or anti-Stokes) frequency, respectively, *N* being the number of molecules, and *n*_*L*_ the number of photons per cm^2^ exciting the process. The resulting electromagnetic enhancement is the product of intensity enhancements |*A*(ν)|^2^ for excitation and scattered field. The cross section for the SERS process *σ*^*SERS*^ is the product of the Raman cross section of the adsorbed molecule and the electromagnetic enhancement. The electromagnetic enhancement, as a consequence of localized plasmon resonances, is frequency dependent. Since the frequency of Raman bands of typical molecular vibrations and the excitation frequency are very similar compared to the spectral width of a typical plasmon resonance, the field enhancement factors *A*(*ν*_*L*_) and *A*(*ν*_*RS*_) in eq 1 are very similar and can be approximated by |*A*(ν)|^4^ for the excitation frequency.

**Figure 1:**
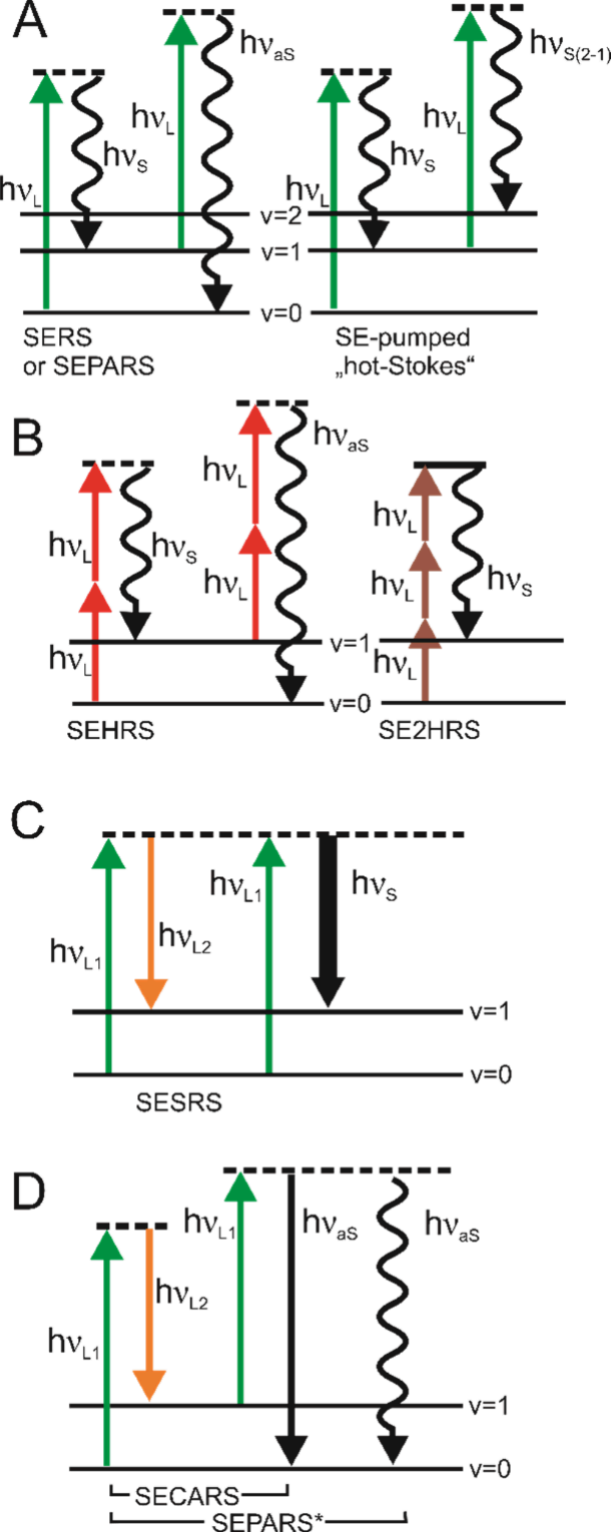
Energy level diagram displaying Raman processes for probing of vibrational transitions of molecules that have been observed to benefit from plasmonic enhancement, indicated by the prefix “SE” in each abbreviation. The energies of the laser(s), Raman Stokes and anti-Stokes scattering are denoted with *hν*_*L*_, *hν*_*S*_, and *hν*_*aS*_, respectively. Excitation can occur to virtual or real electronic excited states, both indicated by the same dashed line. Emission can occur in an incoherent (wiggled arrow) or coherent (straight arrow) process. (A) SERS, SEPARS, and SE-pumped “hot” Stokes process. (B) SEHRS and SE2HRS (C) In SESRS, coherent pumping into the Stokes field is represented by a bold arrow. (D) SECARS is shown together with a SEPARS process that can also be obtained by coherent pumping of the excited vibrational state (denoted with an asterisk). The contributions of the individual fields to the enhancement are summarized in Table 1.

In a nonlinear Raman process, more than one photon contributes to the excitation and therefore the number of Raman scattered photons does not show a linear relationship with the number of photons used to generate them. In such a process, each field is enhanced, and hence nonlinear Raman processes benefit from the electromagnetic field enhancement to much higher extent. Moreover, nonlinear Raman processes advance vibrational spectroscopy, as they can deliver information that is not accessible by linear Raman scattering, such as information on silent modes, sensitive to adsorption geometries, and information on molecule-plasmonic interactions, especially revealed by coherent Raman effects. In the following, we will discuss incoherent (spontaneous) and coherent (stimulated) Raman processes that have been shown to benefit from the electromagnetic surface enhancement, and the information that can be attained from them. Figure 1 displays the incoherent and coherent Raman processes in energy level diagrams.

### Surface Enhanced Incoherent Raman Spectroscopy

#### Surface Enhanced Pumped Anti-Stokes Raman Scattering (SEPARS)

Usually, anti-Stokes signals are much weaker than Stokes signals according to the Boltzmann population of the vibrational levels. In SERS, the surface-enhanced Stokes (S) scattering can measurably populate the first excited vibrational level in addition to the thermal population, which results in unexpectedly high anti-Stokes (aS) signals and increased aS/S signal ratios.^10,11^

Under nonresonant Raman conditions where population of the v = 1 level via molecular electronic excitation can be excluded, population and depopulation of the excited vibrational level can be described by rate equations. Under stationary conditions and in a weakly saturating intensity regime, i.e., , the anti-Stokes signal *n*_*aS*_^*SERS*^ can be estimated according to^10,11^
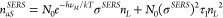
2where *σ*^*SERS*^ is the SERS cross section, τ_1_ is the energy lifetime of the excited vibrational state, *n*_*L*_ is the number of photons per cm^2^ from the excitation laser, *N*_*0*_ is the population of the vibrational ground state, *T* is the temperature, *h* and *k* are the Planck and Boltzmann constants, respectively. The first term describes aS photons related to the thermal population of the v = 1 level, the second term describes the SEPARS photons related to SERS vibrational pumping with an effective cross section *σ*^*SERS*^. SERS pumping in the weakly saturating intensity regime gives rise to a quadratic dependence of the anti-Stokes signal on the excitation intensity. SEPARS can be considered a two-photon process with an effective cross-section *σ*^*SEPARS*^ = (*σ*^*SERS*^)^2^τ_1_ that benefits from enhanced local fields to the power of eight (Table 1). In SEPARS, two excitation photons *hν*_*L*_ interact spontaneously and simultaneously with the same quantum state where *hν*_*S*_ + *hν*_*aS*_ = 2*hν*_*L*_. Pairs of SERS-pumped aS and S photons correlate, as it has been discussed for normal Raman scattering.^12^

From the obtained pumping rate in SEPARS and vibrational life times on the order of picoseconds,^13^ effective nonresonant SERS cross sections can be inferred to be on the order of 10^–16^ cm^2^ per molecule.^10,14^ This is in agreement with SERS enhancement factors on the order of 10^14^, inferred from single molecule SERS spectra that appear at the same signal level as the normal nonresonant Raman signal of 10^14^ methanol molecules.^2^

The observed pumping in nonresonant SERS using continuous-wave (cw) excitation at a relatively low power level also suggests plasmon enhanced *coherent* Raman processes as an efficient way to pump vibrational levels (Figure 1C, SEPARS*).^10^

#### SE-Pumped *v* = 1 to *v* = 2 “Hot” Stokes

SERS vibrational pumping also permits the observation of so-called “hot” molecular transitions from the first (v = 1) to the second (v = 2) excited vibrational levels (Figure 1A, right).^10^ The “hot spectrum” can be extracted from the SERS Stokes spectrum measured in a SERS pumping regime by subtracting the SERS spectrum collected at a low excitation intensity, thermal, regime.^10^ The observed down-shifts for “hot” Raman modes permit to gather information about the anharmonicity of the electronic ground state potentials of a molecule, an information that is usually not accessible from Raman scattering.

#### Surface-Enhanced Hyper-Raman Scattering (SEHRS)

**Figure 2:**
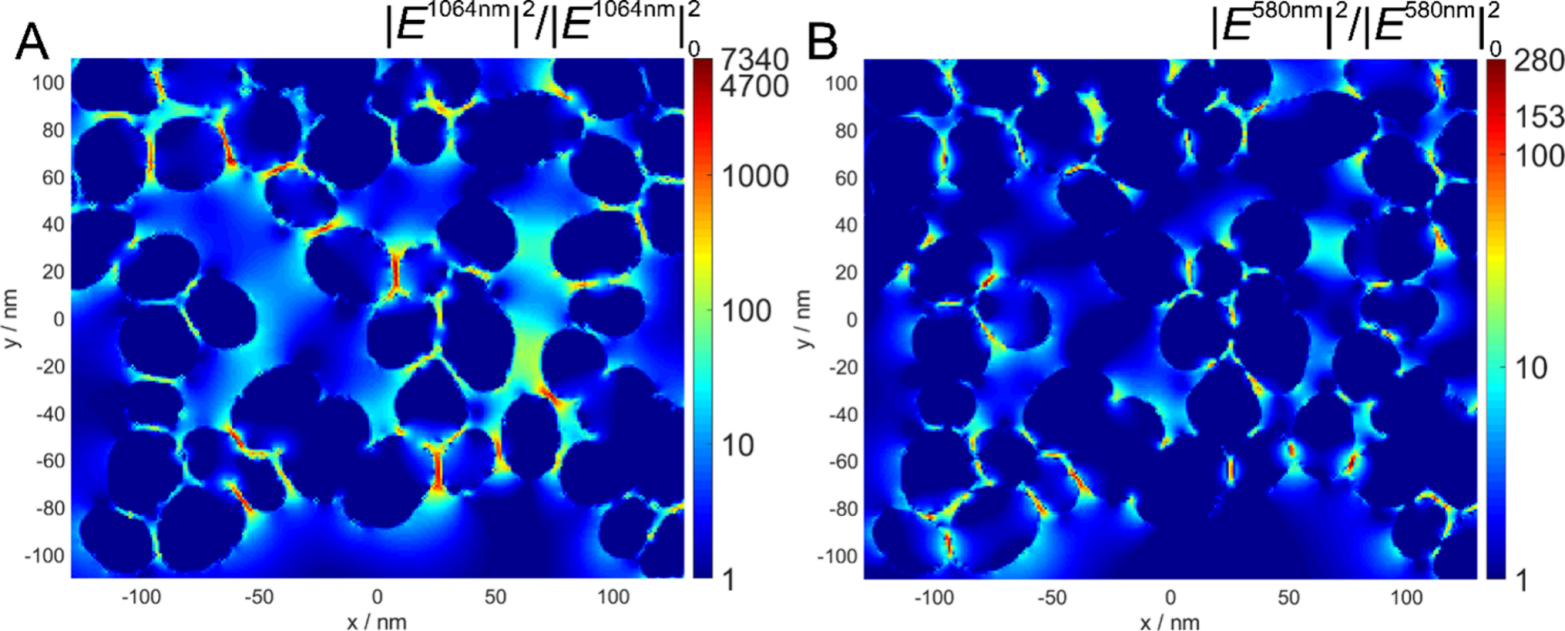
Simulated (3D-FDTD) electric field intensity distribution at (A) an excitation wavelength of 1064 nm, as e.g., used in a hyper Raman experiment and (B) a wavelength shifted with respect to the second harmonic of the excitation, such as a typical Stokes hyper Raman scattering wavelength of 580 nm. The field distribution is shown in the xy-plane for experimentally observed typical arrangements of gold nanoparticles and their aggregates. Note that the spatial coordinates corresponding to maximum field enhancement at 1064 nm differ from those of maximum field enhancement at 580 nm. Reproduced with permission from ref (16). Copyright 2018 American Chemical Society.

In the two-photon excited, incoherent process of hyper Raman scattering (HRS), the scattered radiation is observed near the second harmonic of the excitation wavelength (Figure 1B). Two photons interact simultaneously with the vibrational quantum states based on a change in second-order polarizability (hyperpolarizability) of the molecule. HRS has scattering cross sections on the order of 10^–65^ cm^4^s photon^–1^.^15^ The very weak effect benefits extremely from plasmonic enhancement. The number of SEHRS photons is
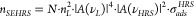
3

with *ν*_*L*_ and *ν*_*HRS*_ being the excitation laser and Raman Stokes (or anti-Stokes) frequency, respectively, *N* being the number of molecules, *n*_*L*_ the number of photons per cm^2^ exciting the process, and σ_*ads*_^*HRS*^ the HRS cross-section of the adsorbed molecule, accounting for the “chemical” enhancement. Due to the dependence on the square of the number of incident photons, the electromagnetic enhancement in SEHRS depends on the enhancement factor of the laser field to fourth power |*A*(*ν*_*L*_)|^4^. The large difference between the frequencies of the excitation laser and the HRS frequency can lead to very different contributions by *A*(*ν*_*L*_) and *A*(*ν*_*HRS*_).^16^ Typically, the frequencies *ν*_*L*_ and *ν*_*HRS*_ are supported by different plasmon resonances of a metal nanostructure. The same nanostructure SEHRS substrate can provide resonances for both fields, though the corresponding “hot spots” have different spatial distribution, e.g. in an array of gold nanoparticles (Figure 2) or also in individual anisotropic nanoparticles, such as gold nanorods.^16^

It should be noted that the chemical contribution to the overall enhancement in SEHRS can be larger than the corresponding chemical enhancement for SERS.^17,18^ Nevertheless, a strong dependence of SEHRS enhancement factors on near-infrared (NIR) excitation wavelengths^19^ indicates that the main contributions to very high SEHRS enhancement by plasmonic support must be key in many experiments. Effective SEHRS cross sections have been quantified in experiments that enabled a direct comparison of SEHRS and SERS signals, in resonant and nonresonant experiments. Effective cross sections in nonresonant SEHRS experiments were estimated to be as high as 10^–46^ – 10^–45^ cm^4^ s photon^–1^,^20^ making SEHRS at least as efficient as a two-photon fluorescence process.

Similar to SERS that can benefit additionally from resonant conditions for the Raman molecule, also SEHRS experiments can utilize two-photon molecular resonances, which enables experiments at the single molecule level,^21^ as well as the use of low pulse energies^22^ or of CW lasers for excitation,^23^ that can be beneficial in many experiments. Resonant SEHRS was shown to be particularly useful to study electronic states of molecules that are inaccessible by one-photon resonant excitation.^24^ Even though in many molecules the resonant SEHRS and SERS spectra are very similar, the SEHRS spectra can contain additional bands, as was shown e.g., for carotene, where infrared-active modes appear.^25^ Resonant SEHRS is a particularly sensitive probe of molecule-plasmonic interactions, because coupling of the molecules to the plasmonic nanostructures modifies the resonance enhancement in the molecules.^25,26^

#### Surface-Enhanced Second Hyper-Raman Scattering (SE2HRS)

It was shown that under molecular resonant conditions together with the plasmonic enhancement, also the second hyperpolarizability of a molecule can be used to study higher-order molecular responses in electronical excited states.^27^ A three-photon excited spontaneous Raman process can occur (Figure 1B, right), using the second hyperpolarizability, resulting in an emission shifted relative to three times the excitation frequency, termed second hyper-Raman scattering (2HRS), so that surface-enhanced 2HRS (SE2HRS) is observed (Figure 1B, right). The fact that such a weak process can be practically exploited illustrates the large influence of the local field enhancement. The enhancement of the excitation field scales with |*A*(*ν*_*L*_)|^6^, with a very large difference in frequency for the laser and Stokes scattered light that contributes an intensity enhancement |*A*(ν_2*HRS*_)|^2^^27^ (Table 1). Also *A*(*ν*_*L*_) and *A*(ν_2*HRS*_) differ due to their very different frequency range and cannot be approximated by a common *A*(ν).

**Table 1:** Enhancement by Local Field *G*_*SE*–*X*_ (*X* Denoting the Respective Raman Process) and Advantages and Limitations of Different Plasmon-Enhanced Nonlinear Raman Processes in Probing Molecules and Nanomaterials

process	enhancement by local field *G*_*S*__*E*__*–*__*X*_	specific advantages	limitations
Incoherent			
SEPARS	*G*_*SEPARS*_ ∝ |*A*(*ν*_*L*_)|^4^·|*A*(*ν*_*S*_)|^2^·|*A*(*ν*_*aS*_)|^2^	signals at the high energy side of the excitation, no fluorescence background;most efficient two-photon process; possibility to infer effective SERS cross sections	very high enhancement factor required
	|*A*(ν)|^8^^a^		
SE-pumped “hot” Stokes	*G*_*SE*–2→1_ ∝ |*A*(*ν*_*L*_)|^4^·|*A*(*ν*_*S*_)|^4^	anharmonicity information from probing higher vibrational levels	very high enhancement factor required;potential superposition by *v* = 0 → *v* = 1 Raman scattering, from which signals have to be retrieved
	|*A*(ν)|^8^^a^		
SEHRS	*G*_*SEHRS*_ ∝ |*A*(*ν*_*L*_)|^4^·|*A*(*ν*_*S*_)|^2^	reveals IR active and silent modes; very sensitive to molecular adsorption geometries; excitation in near-infrared and signals in the visible range; probing of molecular electronic resonances	large difference between ν_L_ and ν_S_; requirements for nanostructures that can support both plasmon resonances
SE2HRS	*G*_*SE*2*HRS*_ ∝ |*A*(*ν*_*L*_)|^6^·|*A*(*ν*_*S*_)|^2^	reveals electronic energy levels	requires support of molecular resonances, requirements for plasmonic spectrum of supporting nanostructures
Coherent			
SESRS	*G*_*SESRS*_ ∝ |*A*(ν_*L*1_)|^4^·|*A*(ν_*L*2_)|^2^·|*A*(*ν*_*S*_)|^2^	potential for microscopy; enables broad band fast imaging, high sensitivity particularly also in biological systems	signals may have to be retrieved from background signals
	|*A*(ν)|^8^^a^		
SECARS	*G*_*SECARS*_ ∝ |*A*(ν_*L*1_)|^2^·|*A*(ν_*L*2_)|^2^·|*A*(ν_*L*1_)|^2^·|*A*(*ν*_*aS*_)|^2^	strong signals at the high energy side of the excitation, no fluorescence background;fast imaging, high sensitivity particularly also in biological systems;line shapes are sensitive to plasmon-molecule interactions; information on vibrational dynamics (energy and phase relaxation) in molecule-plasmonic structures from time-resolved experiments	observation range determined by frequency range of *L2*; dispersive line shape
	|*A*(ν)|^8^^a^		

a Enhancements of different fields *A*(ν) were summarized, assuming they are similar due to similar frequencies ν.

#### Characterization of Molecules at Surfaces with Nonresonant SEHRS

In HRS experiments, vibrational information that is different from that in one-photon excited RS is obtained, due to the effect relying on the hyperpolarizability of the molecule.^28^ For example, all infrared-active modes are also hyper Raman allowed, and also vibrational modes that are “silent” can be hyper Raman allowed.^29^ The great potential of SEHRS to investigate the structure and interaction of molecules on surfaces also in the absence of (two-photon) electronic resonances became clear, when SEHRS spectra of model compounds such as pyridine and bis-pyridyl ethylene were shown to respond strongly to small changes in local adsorbate environments or surface potential.^17,30^ The complementarity of the spectral information and the sensitivity toward small changes in molecular structure and surface interaction have been used to characterize a large variety of organic molecules in their interaction with plasmonic nanostructures, including nucleobases,^31^ amino acids,^32^ and drugs.^33^ The great sensitivity of vibrational modes of the carboxylate group of the molecule 4-mercaptobenzoic acid toward protonation/deprotonation^34^ and binding of uranyl ions,^35^ led to the proposition to use its SEHRS spectrum as probe of pH and in trace analysis, respectively. Different aromatic thiols were also used as “reporter” molecules in nonresonant SEHRS labels for mapping and imaging in living cells.^36^ Similar to two-photon fluorescence, also SEHRS has the advantage that excitation can occur with long wavelengths in the near-infrared, where scattering losses and photodamage are low, making the approach feasible for probing of sensitive materials, e.g. in nanobiophotonics.

**Figure 3:**
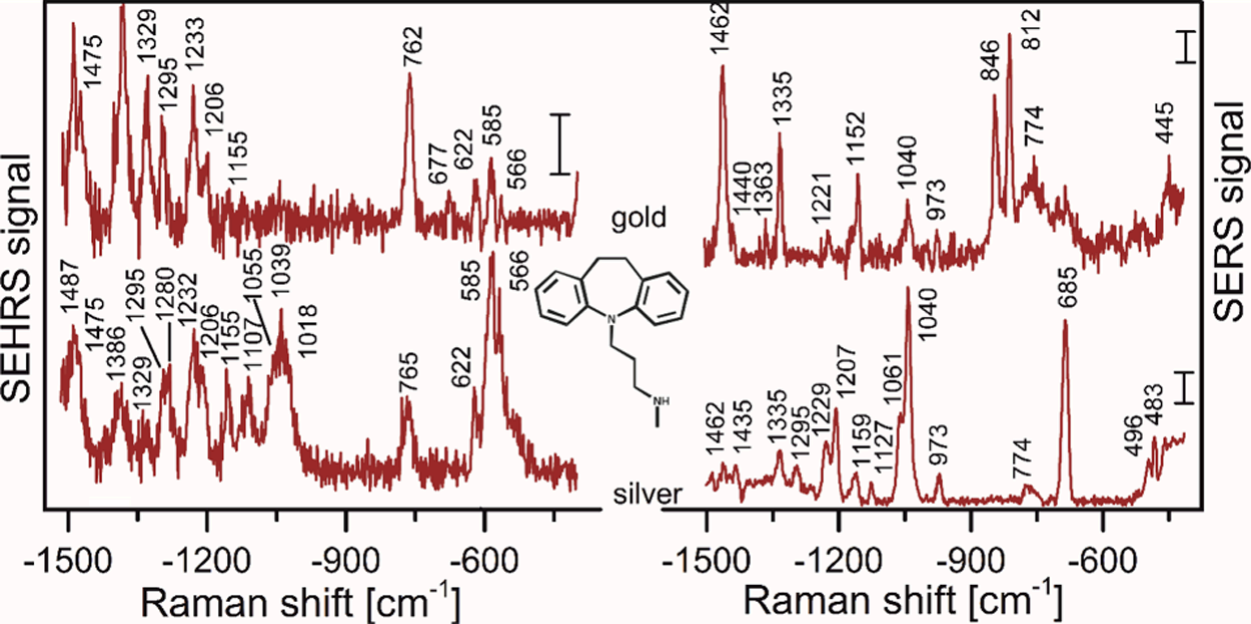
SEHRS (left) and SERS (right) spectra of the antidepressant desipramine obtained with silver and gold nanostructures as denoted. Concentration of the molecule: 9 × 10^–5^ M for gold and 9 × 10^–4^ M for silver nanostructures. SEHRS: Excitation wavelength: 1064 nm, intensity: 2.1 × 10^11^ W cm^–2^, acquisition time: 5 min. SERS Excitation wavelength: 532 nm, laser intensity, 1.2 × 10^10^ W cm^–2^, acquisition time 5 s. Scale bars: SEHRS, 0.1 cps, SERS 50 cps. Adapted with permission from ref (33). Copyright 2017 American Chemical Society.

Figure 3 shows the SEHRS and SERS spectrum of the tricyclic antidepressant drug desipramine. The interaction of desipramine and similar molecules with nanomaterials as well as their biological environment is interesting in the context of drug delivery and theranostics. Despite the different selection rules that are in place for HRS, the SEHRS and SERS spectrum of the molecule are not completely different. Several modes in the fingerprint region are found either in the SEHRS or in the SERS spectrum, or have very different relative intensities in both spectra. Two very intense bands at 846 and 812 cm^–1^ that we assigned to N–C deformation vibrations of the methylaminopropyl side chain, and the 973 cm^–1^ dibenzazepine ring breathing mode are absent from the SEHRS spectrum (Figure 3), similar to their absence in the IR spectrum. In contrast, a strong band at 765 cm^–1^, assigned to a deformation vibration of the −CH groups in the aromatic rings, is particularly enhanced in the SEHRS spectrum, but absent in SERS, and was found in the IR spectrum as well. SEHRS and SERS helped to understand and compare the interaction of the molecule with silver and gold structures, discussed in detail in ref.^33^

**Figure 4:**
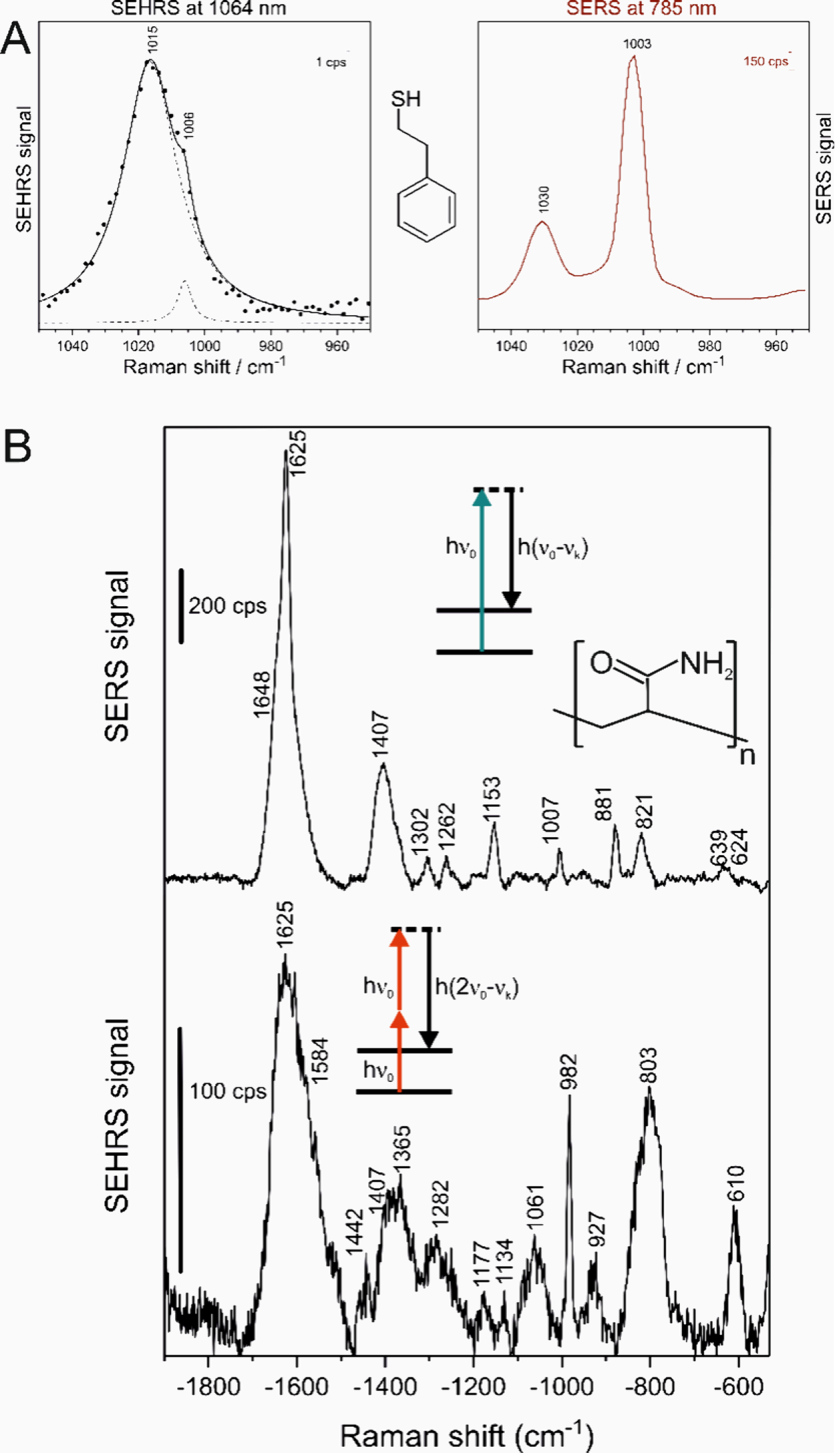
(A) Presence of an additional vibrational band at 1015 cm^–1^ in the SEHRS spectrum (left) of phenylethyl mercaptan on silver nanoparticles as a vibrational mode that is not observed in SERS. Black dots represent experimental data points, the solid line the cumulative fitted curve resulting from the fitted Lorentzian functions (dashed). Reproduced with permission from ref (37). Copyright 2020 American Chemical Society. (B) SEHRS and SERS of polyacrylamide on a silver surface, excited at 1064 and 532 nm, respectively. Several bands in the SEHRS spectrum are assigned to the acrylamide monomer. Reproduced with permission from ref (38). CC-BY license, Copyright 2022, S. Diehn, H. Schlaad, J. Kneipp.

The nonresonant SEHRS spectra of aromatic thiols further underpin the complementary information that can be obtained on molecules when they interact with the surface of nanoparticles. Figure 4A shows the spectrum of phenylethyl mercaptan in the region of its pronounced ring breathing mode, in the SERS spectrum a strong band at 1003 cm^–1^ (Figure 4A, right). In SEHRS, in addition to this mode, a stronger band, assigned to a C–C stretching of the ethyl, combining with a C–S bending vibration and an in-plane ring bending, at 1015 cm^–1^ dominates the spectrum (Figure 4A, left).^37^ This band has low infrared and Raman activity. Moreover, SEHRS was used to sensitively probe the products of surface reactions, as shown for the formation of 2,2′-dimercaptoazobenzene from 2-aminothiophenol in a well-known plasmon-catalyzed oxidation mechanism.^37^ First work on the application of SEHRS to study polymerization in composite nanostructures was reported on the formation of polyacrylamide (PAA) on silver nanoparticles (Figure 4B), where SERS shows bands of PAA, but where the SEHRS spectrum provides evidence that the acrylamide monomer must be present at the surface of the nanoparticles as well.^38^ Moreover, strong enhancement of a band at 982 cm^–1^, an HCCH out-of-plane bending mode that is only weak in SERS, could help to better understand orientation of the polymer on the silver surface based on SEHRS.^38^ The example shows that SEHRS can be useful for the characterization of interfaces in composite materials.

### Surface Enhancement of Coherent Raman Processes

#### Surface Enhanced Stimulated Raman Scattering (SESRS) and Surface Enhanced Coherent Anti-Stokes Raman Scattering (SECARS)

In stimulated or coherent Raman probing, molecular vibrations are coherently driven and also probed by interacting laser fields, resulting in strong coherent Stokes or anti-Stokes signals.

The process is governed by the third order nonlinear susceptibility χ^(3)^, which gives rise to nonlinear polarization terms that oscillate also at Stokes and anti-Stokes frequencies of the excitation lasers.^39^

In stimulated Raman scattering (SRS), the coherent interaction of two lasers ν_*L*1_ (*ν*_*L*_) and ν_*L*2_ (*ν*_*S*_) (fields *E*_*L*_ and *E*_*S*_) whose frequency difference meets the frequency of a molecular vibration gives rise to an increase of intensity in the lower frequency Stokes field (Raman gain), or a depletion in the higher frequency laser field (Raman loss). The nonlinear polarization *P*^*SRS*^, which drives this four-photon process (see also Figure 1C) to create coherent Stokes photons can be expressed as
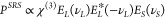
4

During CARS, an excitation laser ν_*L*1_ (*ν*_*L*_) and a Stokes laser ν_*L*2_ (*ν*_*S*_) generate a coherent molecular vibration, where the excitation laser interacts again with this coherent vibration and produces a coherent anti Stokes signal (Figure 1D). The nonlinear polarization *P*^*CARS*^, which is responsible for the generation of coherent anti Stokes photons can be written as
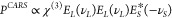
5

Generated SRS and CARS photon numbers *n*_*SRS*_ and *n*_*CARS*_, respectively, show a quadratic dependence on *n*_*L*_ and linear dependence on *n*_*S*_. However, while SRS signals depend on the imaginary part of the complex susceptibility χ^(3)^ and reproduce Lorentzian Raman lines, CARS is determined by |χ^(3)^|^2^, which results in dispersive line shapes. Also, CARS requires phase matching conditions between the interacting fields, while SRS does not depend on the phases of the interacting lasers.^39^

**Figure 5:**
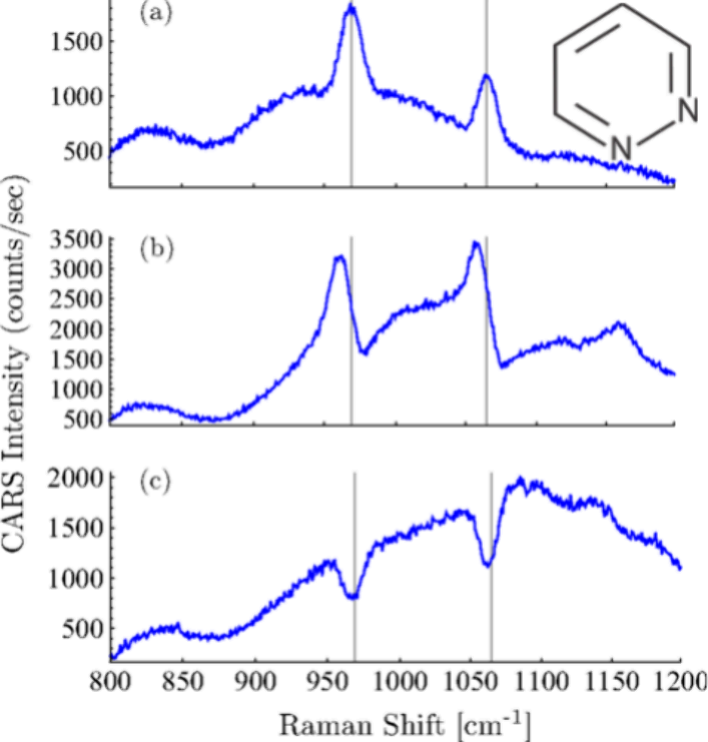
Line shape of spectra revealing the molecule plasmonic interaction in the coherent process of SECARS. Spectra of pyridazine obtained on gold nanoparticle aggregates from different focal spots on a sample. Reused with permission from ref (45). Copyright 2014 American Physical Society.

Due to its strong signals, coherent Raman spectroscopy has demonstrated great potential, and it would be quite challenging to further improve this technique by employing plasmonic enhancement. When SRS and CARS, as four-photon processes, take place in enhanced plasmonic fields, all interacting fields benefit from field enhancement factors *A*(ν) resulting in total intensity enhancement of |*A*(ν)|^8^ (Table 1). The influence of an enhancement of the different fields that yield SECARS is the subject of experimental and theoretical work.^40^ Enhancement in SECARS and SESRS can vary greatly in different experiments.^41−44^ One possible explanation is that in local fields not only field amplitudes are enhanced, but also phases can be influenced by the interaction with plasmon resonances. This does not play a role for the incoherent surface-enhanced Raman processes, but it can show up in phase sensitive processes such as SECARS. Also, the observed signals are superpositions of coherent Stokes or anti-Stokes fields with different phases due their origin from spots with different plasmon resonances.^45^Figure 5 demonstrates the change of SECARS line shapes in a spectrum of pyridazine from additive to dispersive and to subtractive (Figure 5 top to bottom), relative to the four-wave mixing background as the signals are collected from different spots on a layer of aggregated gold nanoparticles.^45^ Effects are also related to the susceptibility χ^(3)^ that includes not only Raman and electronic contributions from the molecule but also resonant and nonresonant contributions from the plasmonic nanostructure and molecule-plasmonic interactions. This is further supported by the observation of strong changes in line shapes in SESRS spectra, when frequencies of the interacting fields are shifted relative to the plasmon resonance.^46^ The highly localized interaction of different fields that shows in the phase sensitive coherent processes has the potential to become a sensitive probe of plasmon-molecule/material interaction in hybrid materials, e.g. in the design of systems for efficient light harvesting. Provided that the interaction of electromagnetic fields and their role in the SECARS process of a system is understood, additional chemical effects can be used as well in order to create further enhancement. As an example, 2-dimensional MoS_2_ nanocrystals were shown to provide a very large chemical enhancement of CARS due to charge transfer states and resonant excitation.^47^ On top of the well-characterized plasmonic field enhancement, chemical enhancement based on charge transfer could be a sensitive probe for studying surface processes in catalysis or spectral sensitization.

Surface-enhanced coherent Raman scattering and also its applications are discussed in comprehensive reviews.^42,43^ SECARS provides particularly favorable conditions for molecular imaging with signal strengths several orders of magnitude higher than signals in “normal” CARS^41,44^ and was, e.g., used for the detection of SERS labels in complex bioenvironments^48^ and recently shown to enable the acquisition of millions of spectra in wide-field SECARS microscopy.^49^ Plasmon enhanced SRS microscopy can be carried out with single-molecule sensitivity.^50^ By exploiting plasmonic Fano resonances for creating high local fields, SECARS has demonstrated single molecule spectral sensitivity.^51^

When used in pump–probe experiments, surface-enhanced coherent Raman scattering provides exciting capabilities, as it can combine high spatial and temporal resolution with high sensitivity and provides comprehensive insight into molecular dynamics and chemical processes on the surface of plasmonic structures.^43^ The use of optimized plasmonic support enables pump–probe coherent Raman experiments at single molecule level and on individual nanostructures.^42^

The adaptation of a method developed for extracting information on energy and phase relaxation of molecular vibrations based on the measurement of coherent and incoherent anti-Stokes signals^13^ allows to study the molecular vibrational decay of a molecule in a plasmonic cavity.^52^ In this time-resolved SECARS study,^52^ the molecular excitation is coherently excited by two lasers. A third laser probes the excitation at different delay times. From the time behavior of the coherent SECARS signal and the spontaneous incoherent SEPARS signal (cf. Figure 1D), phase and energy decay times, respectively, can be directly inferred. The study shows that for a molecule in a plasmonic local field the vibrational dephasing rates of the molecule are accelerated with increasing illumination intensity, while the energy relaxation remains constant.^52^ It sets an example on the wealth of information that can be obtained on molecule-plasmon interactions based on time-resolved coherent Raman experiments, and on potential ways to control important physical properties of plasmonic materials.

## Conclusions

Nonlinear Raman scattering performed in plasmonically enhanced local field provides exciting capabilities for structurally sensitive vibrational probing and imaging and particularly for probing molecule-plasmonic interactions. Very weak spontaneous nonlinear Raman scattering, excited by two or even three photons becomes usable toward an advanced spectroscopic characterization of molecular structure and interactions at nanostructure surfaces and in composite nanomaterials. The complementary spectral information that is obtained in SEHRS adds insight, e.g., on different types of adsorbates, changes in their local molecular environment, and modifications due to photochemical reactions. Work with biomaterials and with products of chemical reactions, such as polymerization reactions and plasmon-supported transformations illustrates the potential of the approach in different nanomaterials-related fields where optimized light-matter interactions are important.

In addition to several practical advantages known for multiphoton excitation, excitation off-resonance with electronic transitions in molecules is attractive, due to nonselective probing. In excitation conditions that are in (multiphoton) resonance with a molecule, electronic states can be probed that are not accessible by one-photon excitation. Raman probing of excited vibrational levels by strong nonresonant SERS pumping can give information on molecule anharmonicity that is connected to important chemical and physical properties of materials, including reactivity and heat transfer.

Coherent, stimulated Raman processes do not necessarily have to rely on plasmonic enhancement in order to achieve reasonable signal levels in an experiment, but also here, improved signals due to plasmonic support will enable molecular sensing and particularly also fast imaging. More importantly, the interaction of coherent fields is very sensitive to molecule-plasmon interactions and provides SECARS and SESRS with rich qualitative information on molecule/material-plasmon interactions. Such interactions reveal themselves by altered line shapes. Time-resolved CARS can directly probe their dynamics. Although this discussion focused on the local fields due to their obvious role in enhancement, a better understanding and control of the electromagnetic fields will of course also help an elucidation of electronic (chemical) interactions and enable tailoring of hybrid plasmonic nanomaterials with optimized optical properties.

## Abbreviations

aS: anti-Stokes
CARS: coherent anti-Stokes Raman scattering
HRS: hyper-Raman scattering
RS: Raman scattering
RRS: resonant Raman scattering
S: Stokes
SERS: surface enhanced Raman scattering
SEHRS: surface enhanced hyper-Raman scattering
SECARS: surface enhanced coherent anti-Stokes Raman scattering
SEPARS: surface enhanced pumped anti-Stokes Raman scattering
SESRS: surface enhanced stimulated Raman scattering
